# Supplementary material for: Are carbon emissions trading and green financial instruments synergistic? -Comprehensive quantitative research based on content analysis
Source: PLoS One. 2024 Mar 7;19(3):e0298601. doi: 10.1371/journal.pone.0298601 (PMC10919601; doi:10.1371/journal.pone.0298601)
Supplement: S1 Data — (ZIP) [file pone.0298601.s001.zip › Data/地级市Z_F工作报告.zip!/║╙▒▒/╨╧╠¿╩╨/└┤╫╘╣1⁄2╓┌║┼í╛┬φ┐╦╩2╛▌═°í┐_2019.docx]

各位代表：

　　现在，我代表邢台市人民政府向大会作工作报告，请予审议，并请各位政协委员和列席的同志提出意见。

　　一、2018年工作回顾

　　过去的一年，是全面贯彻落实党的十九大精神开局之年，也是全市上下攻坚克难、砥砺奋进的一年。一年来，我们坚持以习近平新时代中国特色社会主义思想为指导，树牢“四个意识”，践行“两个维护”，坚持稳中求进工作总基调，贯彻落实新发展理念，坚决贯彻省委、省政府和市委决策部署，奋力推进创新发展、绿色发展、高质量发展，较好完成了市十五届人大三次会议确定的目标任务。

　　——综合实力在复杂形势下迈上新台阶。坚持把发展作为第一要务，牢牢把握“稳、进、好、准、度”，一般公共预算收入完成149亿元，同比增长17.6%，连续两年增速全省第一；生产总值增长7.0%，规模以上工业增加值增长6.2%，固定资产投资增长6.1%，社会消费品零售总额增长9.4%，主要经济指标增速均高于全省平均水平。

　　——产业层级在动能转换中实现新提升。深化供给侧结构性改革，破立结合、消长并重，三次产业结构由11.8∶45.6∶42.6调整为12.3∶40.8∶46.9，第三产业占比首次超过第二产业、对经济增长的贡献率达到59.7%。装备制造业对规模以上工业增加值的贡献率达到47.1%，高新技术产业增加值增速高于规模以上工业5.1个百分点，预计单位生产总值能耗、水耗分别下降5.5%、3.3%，削减煤炭消费46万吨。

　　——生态治理在巨大压力下取得新成效。以前所未有的决心和力度打好污染防治攻坚战，以时保天、以月保年，PM2.5平均浓度达到69微克/立方米，较2013年下降56.9%，优良天数160天，比2013年增加122天。扎实推进水污染治理百日攻坚，城市黑臭水体基本消除，消纳江水3.4亿立方米，狗头泉、百泉时隔18年再展碧波。完成营造林50.82万亩，预计净增森林覆盖率1个百分点以上。

　　——民生事业在共建共享中得到新发展。始终将增进民生福祉作为发展的根本目的，财政用于民生支出393.3亿元，占一般公共预算支出的79.7%。城镇、农村居民人均可支配收入预计分别达到28142元、11879元，分别增长7.5%、8%。新开工棚户区改造住房2.5万套，基本建成1.76万套。居民消费价格涨幅控制在2.5%以内，常住人口城镇化率预计达到52.87%。

　　一年来，我们主要抓了以下工作：

　　（一）着力发展有特色的产业，质量效益同步提高。传统产业以搬促提、加快转型升级，新兴产业扩能增量、不断发展壮大，产业结构实现新优化。坚定不移去产能，压减退出炼铁50万吨、煤炭210万吨、焦炭70万吨、平板玻璃250万重量箱，旭阳焦化、建滔化工冷炉封停5座焦炉，涉及焦炭产能220万吨。出台实施工业转型升级、战略性新兴产业等系列三年行动计划，明确了高质量发展的路径举措。投资80亿元的中钢邢机搬迁升级项目全面开工，工作专班先后召开56次例会，以政策创新撬动市场投资，铸就了邢台历史上最大的工业项目，一招走活了城市发展、生态建设、产业转型三步棋，伴随邢台工业成长“一甲子”的中钢邢机迎来了凤凰涅槃。晶澳实现美国退市回归，太阳能组件项目列入2018年国家级智能制造示范项目，欧耐、金泰成等5家企业被认定为全国第三批绿色工厂，多氟多红星、领途“第三工厂”等新能源汽车项目进展顺利。清河羊绒、隆尧铸铁炊具等特色产业引入工业设计，产品附加值和市场竞争力进一步提升，任县、广宗、临城等6个县（市）获评河北特色产业名县。大力实施乡村振兴战略，深化农业供给侧结构性改革，粮食总产466.5万吨，今麦郎、金沙河、玉锋等一批新项目相继投产，小麦、玉米加工能力分别达到全市年产量的2.8倍、2.2倍，农业产业化经营率预计达到70.7%，46个项目入选省“四个一百工程”、数量全省第一，桥西区完成全国农村集体产权制度改革试点任务，临城核桃、巨鹿金银花入选首届中国农民丰收节百个农产品品牌名单，中国优质小麦产业发展大会、全国小麦节水保优现场会、河北省农业产业化暨奶业振兴工作会议、河北省首届中国农民丰收节活动在我市举办。串联9个景区的百里旅游专线抗大路顺利通车，旅游业总收入预计增长21%。万达广场开业首日客流30万人次。新增3家新三板、5家石交所挂牌企业，1家银行、4家保险公司在我市新设分支机构。

　　（二）着力集聚高质量的项目，发展后劲持续增强。把项目建设作为高质量发展的突破口和着力点，实施项目建设“月通报、季度擂台赛、半年观摩拉练”，连续18周利用周末开展“周周促”活动，在全市激发增投资、上项目的浓厚氛围。46个项目列入省重点、数量全省第二，200个市重点项目中，战略性新兴产业和服务业项目占到47%，石药生物科技产业园、中伟卓特液压驱动系统、浩联油气热能输送管道、熙甄高档羊绒超薄面料等85个项目竣工投产，省市重点项目完成投资400亿元。持续深化开发区改革，全面提升园区承载能力，加快“新九通一平”建设，新建成7座开发区污水处理厂，邢台经开区、清河经开区获评全省开放发展十佳开发区，滏阳高新区更名为邢台高新区。坚定不移走开放合作之路，先后举办特色食品、羊绒、自行车等一系列对接会、博览会，叫响邢台品牌，推动开放共赢。深度融入京津冀协同发展，引入京津优质项目61个。投资40亿元的好孩子“甲天下”项目签约落地。与华为公司开展战略合作，投资10亿元共建大数据产业园，“云上邢台”全面上线。积极应对中美贸易摩擦影响，发展外贸新业态新模式，出口总值增长12%，外商直接投资增长5%。主动融入“一带一路”建设，好望角物流在乌兹别克斯坦建设河北工业园项目，德龙钢铁等6家企业实现海外布局。

　　（三）着力营造有活力的环境，改革创新全面发力。坚持以邢台态度提升邢台速度，扎实开展“双创双服”活动，持续深化“放管服”改革，全面衔接国务院和省政府取消调整行政权力事项，向市开发区“点菜式”下放权力333项，在全省率先实现“五十二证合一”，市级政务服务事项网上可办率达到96%，县级达到85%，企业开办时间平均压减至5个工作日以内。创新实施“两不见面”改革，在国务院第五次大督查中树为典型案例，央视《经济半小时》栏目专题报道。在深化金融改革方面，着力优化贷款投向结构，企业贷款占到全市新增贷款的65.58%，较上年增加246亿元，创新开展“金融产品+企业需求”双推送，向小微企业新发放贷款73.2亿元。全面落实减税降费政策，预计减税41亿元、降费1.65亿元，让企业享受到真金白银的改革红利。市重点企业服务办公室帮助企业解决60多件发展难题，纳税百强企业上缴税金增长23.7%。新登记市场主体增长20.1%，万人拥有市场主体突破700户。深化国企国资改革，61家市属企业遗留问题基本解决，1.7万余名职工得到妥善安置，涉及10万余企业职工的“三供一业”分离移交任务基本完成。巨鹿医养结合、威县综合改革入选全国“改革开放40周年地方改革创新40案例”。市级机构改革基本完成，县级机构改革稳步推进。大力实施创新驱动战略，新增省级科技创新平台20家、省级院士工作站3家，预计新增科技型中小企业超过1000家、高新技术企业85家，万人发明专利拥有量达到1.2件，技术合同交易额增长45%，中国驰名商标达到41件。

　　（四）着力建设有特点的城市，品质价值显著提升。聚焦群众关心的城市堵点、难点，持续加大投入力度，实施7大类191项城建重点工程，城市变得更新更美更舒朗。太行山高速邢台段在全省率先通车，大外环实现部分通车。邢东新区发展框架全面拉开，金融中心开工建设，规划展馆主体完工，邢台精英中学投入使用。主城区新改建道路58条，贯通断头路28条，育英街地道桥顺利开通，投资4.4亿元的莲池大街铁路上跨桥建成通车。完成市区20条小街巷整治提升，敷设供水管网52.8公里、供热管网23.2公里、燃气管网17公里，主要街道基本实现雨污分流，投资4.5亿元的市第二污水处理厂投入运行。环城水系加快建设，围寨河、小黄河下游综合治理完工通水，东关河建设全线展开，省园博园土方工程、主园路基本完工，新建游园30处，新增绿地250万平方米，生态基底日趋完善。以国家级文明城市创建为抓手，全民动员、全员参与，城市管理水平、文明程度进一步提升。“一区三边”拆违拆迁801.6万平方米，256项房地产遗留问题全部解决，171个老旧小区完成改造，新建5处林荫式停车场，施划1.75万个停车位，首个大型生活垃圾压缩转运站建成运行。农村人居环境整治三年行动扎实开展，清理积存垃圾446万立方米，改造农村厕所30.2万座。县城建设三年行动顺利推进，527项重点工程完成投资200亿元、增长24%。内丘苹果小镇、柏乡牡丹小镇等8个小镇入选省百个旅游特色小镇。

　　（五）着力破解制约性难题，三大攻坚战取得实效。主动防范化解重大风险，抓好地方债务存量化解和增量控制，加强金融风险监管和预警处置，严厉打击非法集资、违规融资担保等行为，清理整顿非法经营机构87家。打好精准脱贫攻坚战，市县财政用于扶贫支出增长56.3%，探索建立“三包一联”精准帮扶机制，实施产业扶贫项目270个，改造农村危房4910户，完成易地扶贫搬迁347户、901人，贫困群众全部纳入低保范围，医保报销比例提高到95%。威县、内丘等6个县实现脱贫摘帽，214个贫困村脱贫出列，5.49万贫困人口稳定脱贫。持续推进污染防治，实施工业企业深度治理工程77项，整治“散乱污”企业876家，淘汰35蒸吨及以下燃煤锅炉59台，低氮燃烧改造锅炉671台，我市入选第二批中央财政支持北方地区冬季清洁取暖试点城市，新增“双代”清洁取暖16万户；落实“河长制”要求，“一河一策”开展系列专项行动，封堵非法排污口925个，清理河道垃圾38.3万立方米，拆除沿途旱厕271个，扎实做好中央、省环保督察“回头看”问题整改，生态环境质量得到进一步提升。

　　（六）着力抓好有温度的民生，人民生活日益改善。践行以人民为中心的发展思想，把目光聚焦到群众烦心事，把精力聚焦到民生细微处。强化社会保障，城乡最低生活保障标准分别提高10.3%和9.1%，医保补助提高到490元/年，基础养老金最低标准提高到108元/月。提高教育水平，每季度观摩调度主城区基础教育三年攻坚行动，项目学校完工23所、在建39所，南宫、南和、桥西区等7个县（市、区）通过国家义务教育发展基本均衡县验收，宁晋、邢台县入围国家级农村职业教育和成人教育示范县。全力做好就业服务，新增城镇就业7.54万人，转移农村劳动力7.57万人次，城镇登记失业率3.46%。打造健康邢台，医改工作扎实推进，市三院门诊楼建成投用，内丘获评河北省中医药强县。连续办好国际公路自行车大赛和市旅发大会，首倡太行山文化带建设交流会，城市知名度进一步提升。话剧剧本《郭守敬》、文艺栏目《邢台记忆》等7部作品入选省文艺振兴奖。太行新愚公李保国被党中央表彰为“百名改革开放杰出贡献者”。严守国家机密的退役军人吴洪甫荣登“中国好人榜”，被评为全国“最美退役军人”。邢台姑娘尹军花勇夺亚运会女子拳击金牌。全市村（社区）“两委”换届工作圆满完成。深入开展扫黑除恶专项斗争，持续推进“创净土”系列行动，严厉打击各类违法犯罪，开展安全生产“双清双提”，加大食品药品安全监管力度，社会大局保持和谐稳定，群众的安全感进一步提升。

　　同时，双拥共建、民族宗教、新闻广电、外事侨务、人民防空、气象地震、史志档案、邮政通信、审计统计、公积金、老龄妇幼、残疾人事业、援疆援藏等工作都取得新成绩。一年来，我市承办省级以上现场会38次，在国家和省会议上作典型发言48次，37项工作获得国家和省领导的批示肯定。

　　一年来，我们认真学习贯彻习近平新时代中国特色社会主义思想和党的十九大精神，坚决落实全面从严治党战略部署，认真做好中央和省委巡视反馈意见整改，严格落实中央八项规定及其实施细则精神，深入开展“一问责八清理”、纠正“四风”和作风纪律专项整治，持之以恒正风肃纪，政府公信力执行力不断提升。忠诚履行宪法和法律赋予的职责，持续完善依法科学民主决策机制，积极推进地方立法。自觉接受人大法律监督、工作监督和政协民主监督，办理人大议案建议351件、政协提案904件，办结率100%。政府职能转变、作风建设取得扎实成效。

　　各位代表！回顾2018年，我们面临的形势比预计的更为严峻，遇到的困难比原想的更为复杂，走过的道路比以往更为不平凡，取得的成绩比以往更为不容易。这一年，我们顶住了压力挑战，以改革创新干出了一批实事，以责任担当解决了一批难事，以使命初心回应了群众的新期待。全市人民发自内心感恩习近平总书记、感激党中央，正是因为习近平总书记对河北知之深、爱之切，才有了新时代邢台发展的新局面，我们坚决以办好邢台事情、加快高质量发展的实际行动，维护核心、拥戴核心、捍卫核心，做习近平新时代中国特色社会主义思想坚定的信仰者、有力的传播者、忠诚的践行者。

　　各位代表！新时代是奋斗者的时代，幸福都是奋斗出来的。在2018年的历程中，许多普普通通的邢台人为了“邢台更行”而默默付出。我们不能忘记，广大企业家和职工队伍，积极响应号召、履行社会责任，顶住了经济下行压力，在推动产业转型升级、加快高质量发展中闯出了一条新路，邢台发展的底气更足了。我们不能忘记，在脱贫攻坚一线，驻村干部、第一书记、帮扶工作队员，舍小家、为大家，奔走田间地头，倾听家长里短，与乡亲们共谋脱贫之路，工作很投入，很给力。我们不能忘记，面对大气污染治理的严峻形势，全市上下协力攻坚，广大干部职工和基层网格员坚守工作岗位，奋战至元旦零时不放松，圆满完成目标任务。我们不能忘记，在自行车大赛、旅发大会等重大活动期间，基层干部甘于奉献，安保队伍威武文明，观赛群众热情高涨，文艺展演和啦啦队神采飞扬，“再使累慌也不觉得使累慌”展现了邢台人民的朴实和善良。我们不能忘记，全市上下热火朝天抓创城、搞建设的难忘画面，城市“建造师”挥汗如雨、街头“扫帚官”辛勤劳动、志愿者“小红帽”传递文明，广大市民对修路限行带来的耽误接孩子、看老人以及工作生活上的不便，给予了最大支持和包容，点点滴滴汇聚起城市面貌的日新月异。我们不能忘记，与歹徒搏斗时身负重伤仍冲锋在前的公安干警，救护车侧翻时齐心救人的公交司乘群体，坚守太行山深处30年的最美教师夫妇，深入一线了解民意社情的人大代表、政协委员，还有广大科技工作者、医护人员、农民兄弟、快递小哥、出租车司机、媒体网民朋友和千千万万的普通劳动者，用辛劳拱卫着邢台的平安，用汗水浇灌着邢台的成长。大美无华、大爱无疆，我们为这些美好生活的守护者、创造者点赞！

　　各位代表！奋斗充满艰辛、成就令人鼓舞。我们所作的每一项工作、取得的每一点成绩，都得益于习近平新时代中国特色社会主义思想的引航定向，得益于省委、省政府和市委的坚强领导，得益于人大代表、政协委员以及社会各界的关心支持，得益于全市人民的共同努力和不懈奋斗。在此，我代表市人民政府，向全市人民，向人大代表、政协委员，向各民主党派、工商联、无党派人士和人民团体，向驻市部队、武警官兵、政法干警和驻市各单位，向所有关心支持邢台发展的各位老领导、老同志，向广大企业家以及海内外朋友，表示崇高的敬意和衷心的感谢！

　　在看到成绩的同时，我们也清醒认识到，目前邢台正处于转型升级、爬坡过坎的关键时期，还有很多深层次的矛盾和问题。一是经济发展与人口大市地位不相匹配，总量指标依然落后，人均指标大多排在全省末位。民生支出不断增长，财政收入不能完全满足日益增长的支出需要，收支矛盾亟待破解。二是产业层级总体偏低，传统产业大而不强，链条短、集约差、低端化、同质化的现象还不同程度存在；新兴产业发展较快，但规模较小，短期难以填补去产能的空缺。三是大气污染治理空间日渐收窄，水污染治理还存在差距，部分区域、部分行业生态环境问题依然突出。四是教育、医疗、养老、文体等基本公共服务供给还不充分，人民群众还有不满意的地方，加速新型城镇化进程仍然是今后一个时期的紧迫任务。五是风险隐患不容忽视，部分县（市、区）政府债务偏高，安全生产基础还不够牢固，社会治理面临一系列新挑战新要求。此外，政府职能转变还不到位，政府工作人员的法治思维、专业素养、开放意识需要进一步提升，不正之风和腐败问题仍有发生。2018年《政府工作报告》确定的233项重点任务，还有5项没有完成。对此，我们一定下大力解决。

　　各位代表！一年来的艰苦奋斗，让我们深切感受到，低头靠勇气，抬头靠实力，越是经济形势复杂严峻，越要坚定信心决心，拿出不气馁、不服输的韧劲，咬紧牙关、拼搏进取，在探索中找出路、想办法，转危机为契机，变难点为亮点。越是供给侧结构性矛盾凸显，越要坚持新发展理念，把高质量发展作为解决一切问题的基础和关键，保持战略定力、毅力和耐力，横下一条心，持之以恒、久久为功。越是中流浪急、半山坡陡之时，越要坚持全面深化改革、扩大高水平开放，坚决破除制约高质量发展的思想束缚和制度藩篱，进一步解放思想、创新思路，形成富有活力的体制机制。越是临近全面建成小康社会目标，越要增强忧患意识，健全各方面风险防控化解机制，严防“黑天鹅”“灰犀牛”，确保经济平稳运行、社会和谐稳定。越是面临多重压力综合考验，越要坚持以人民为中心的发展思想，团结和依靠人民，激发人民群众的创造伟力，在共建共享中让人民群众有更多、更直接、更实在的获得感。我们坚信，只要沿着习近平总书记指引的方向，坚定不移走下去，就一定能够在新时代谱写新篇章、夺取新胜利！

　　二、2019年工作安排

　　今年是新中国成立70周年，是全面建成小康社会、实现第一个百年奋斗目标的关键之年，做好全年工作至关重要。当今世界面临百年未有之大变局，我国发展仍处于并将长期处于重要战略机遇期，习近平总书记亲自谋划、亲自推动的京津冀协同发展、雄安新区规划建设、冬奥会筹办在河北同期实施，使我省重大机遇叠加交汇，形成了强大的发展动力和独特的发展优势。我们要深刻领会重要战略机遇期新内涵，抢抓加快经济结构优化升级、提升科技创新能力、改革开放、加快绿色发展、参与全球经济治理体系变革带来的五大新机遇，全力推动重大国家战略和国家大事在邢台落实落地，把握好邢台发展的阶段性特征，强化战略思维、系统思维、辩证思维，因时而动，顺势而为，变短板为潜力，化挑战为机遇，努力实现变道超车、协同发展。我们必须坚持解放思想、勇于突破，以敢闯敢试、敢为人先的改革精神突破桎梏，加快推动创新发展。我们必须坚持生态优先、标本兼治，在治理污染、修复生态中加快营造良好人居环境，着力推动绿色发展。我们必须坚持质量第一、效益优先，在质的大幅提升中实现量的有效增长，全面推动高质量发展。

　　今年政府工作的总体要求是：坚持以习近平新时代中国特色社会主义思想为指导，全面贯彻党的十九大和十九届二中、三中全会精神，深入落实中央经济工作会议、省委九届八次全会、市委九届六次全会精神，统筹推进“五位一体”总体布局，协调推进“四个全面”战略布局，坚持稳中求进工作总基调，坚持新发展理念，坚持推进高质量发展，坚持以供给侧结构性改革为主线，坚持“三六八九”基本思路，聚焦“巩固、增强、提升、畅通”八字方针，统筹推进稳增长、促改革、调结构、治污染、惠民生、防风险各项工作，保持经济运行在合理区间，保持社会大局和谐稳定，保持经济发展持续向好，为全面建成小康社会收官打下决定性基础，以优异成绩庆祝中华人民共和国成立70周年。

　　今年经济社会发展的主要预期目标是：生产总值增长7%左右，一般公共预算收入增长7%，规模以上工业增加值增长6%左右，固定资产投资增长6.5%左右，社会消费品零售总额增长9.5%，实际利用外资增长5%，城镇登记失业率控制在4.5%以内；城镇、农村居民人均可支配收入分别增长7.5%、8.5%；居民消费价格指数涨幅控制在3%左右；森林覆盖率提高1个百分点，PM2.5平均浓度下降率、万元生产总值能耗下降率以及化学需氧量、二氧化硫、氨氮、氮氧化物减排完成省下达目标任务。指标计划略高于全省平均水平，既充分体现了贯彻中央和省决策部署的信心和决心，又立足于我市现有经济基础和产业支撑，统筹了总量增长和结构优化，体现了稳中求进要求和高质量发展导向。

　　做好全年工作，重点是抓好以下七个方面：

　　（一）以深化供给侧结构性改革为主线，推动制造业高质量发展。贯彻落实“巩固、增强、提升、畅通”八字方针要求，强力实施系列三年行动计划，依托完备的制造业基础，着力强链、补链、延链，重点做强先进装备制造、健康食品、新材料三大优势主导产业，壮大新能源和新能源汽车等新兴产业，提升若干特色产业集群，加快构建“3+1+N”产业体系。坚定不移化解过剩产能。执行更加严格的质量、环保、能耗、水耗、安全等标准，综合运用市场化、法治化手段，推动总量去产能向结构去产能转变。严格落实省下达的炼铁、焦炭、玻璃等行业去产能任务。深入细致做好去产能职工安置工作，妥善处理各类遗留问题，确保职工合法权益。做大做强优势主导产业。深入开展“千企转型”行动，大力实施传统产业技术改造，技改投资增长3%以上。以建设华北地区重要的先进装备制造基地为目标，加快中钢邢机、邢钢搬迁升级步伐，大力发展农机、冶金、矿山、石油钻采装备制造，在节能环保、轨道交通等高端装备制造领域实现新突破。加快健康食品产业发展，支持今麦郎研发基地及创新制造、君乐宝液体乳及饮料等项目建设，光明百万头生猪肉联加工一体化生产基地项目建成投产。壮大中科鑫宝、晟华溅射靶材等新材料企业，打造全省重要的先进结构和新型功能材料产业基地。推动新兴产业提质增效。深入实施战略性新兴产业三年行动计划，推进战略性新兴产业基地和“双创”示范基地建设，规模以上高新技术产业增加值增长10%左右。华菱长征新能源（LNG）中重卡启动四大工艺建设，领途“第三工厂”新能源车下线。晶龙单晶硅二期、晶澳2GW太阳能组件扩能项目建成投产，新建投资50亿元的晶龙新能源产业园项目，打造全球重要光伏产业基地。支持金后盾等军民融合企业发展，加快康德无人飞行器及航空零部件等项目建设，争创省级军民融合产业示范园，军民融合企业达到100家左右。促进先进制造业与现代服务业深度融合。培育引进更多工业设计机构和应用平台，以工业设计提升特色产业品质价值，打造“专精特新”隐形冠军。促进“互联网＋”和“＋互联网”相协同，加快发展高端研发、智慧物流等现代服务业，积极培育北斗应用、虚拟现实等未来产业。深入实施制造强市、质量强市和标准化战略，大力支持企业主导或参与各类标准制修订，推动标准、质量、品牌、信誉联动提升，加快实现邢台制造向邢台创造、邢台速度向邢台质量、邢台产品向邢台品牌转变。加快县域经济发展。制定激励县区经济发展的财政政策，强化科技创新，加强配套协作，推动县域经济特色化、特色经济产业化、产业经济集群化，力争全年生产总值超百亿元的县（市、区）达到10个以上。

　　（二）以激发有效需求为导向，确保经济运行在合理区间。稳是大局，稳是主基调。充分发挥投资对优化供给结构的关键性作用，消费对经济发展的基础性作用，促进民营经济健康发展，为经济平稳运行提供有力支撑。开展“重点项目建设质量年”活动。强化高端高质项目引领，推进省级39个、市级300个重点项目，继续实行“周周促、月通报、季度擂台赛、半年观摩拉练”，研究制定土地资源供应的差别化管理机制和准入政策，引导优质资源向重点项目、重点产业集中，土地、资金等要素向能开工、能竣工的项目倾斜，把有限资源用在最能推动高质量发展的刀刃上。扩大消费新需求。顺应消费升级新趋势，着力增加优质产品和服务供给。办好市旅发大会，支持邢台大峡谷、扁鹊庙争创5A级景区，抓住太行山高速通车契机，加快西部山区特色农业、文化旅游、中医药康养产业“一路三带”建设。推进电子商务进农村综合示范项目，加强全市电子商务综合服务平台建设。规划建设面包烘焙小镇，鹊山水世界建成开业。旅游业总收入达到350亿元。加强市内企业间的相互采购，搭建常态化产品对接平台，鼓励政府采购在同质同价条件下优先使用本地产品。毫不动摇鼓励支持引导民营经济发展。深入开展减税降费专项行动，力争再降低实体经济成本17亿元。继续实施“双推送”机制，组织开展企业评金融活动，建立民营企业纾困基金，积极帮助企业拓宽融资渠道。加快晶澳上市步伐，推动根力多、精晶药业、今麦郎、亿利在沪深交易所上市，中航上大在科创板上市，欧耐在新三板挂牌。强化企业家教育培训，搭建学习交流平台，提升企业家综合素质和企业核心竞争力。民营企业和民营企业家是我们的自己人，要高看厚爱、真心呵护、倾力支持，依法保护企业家人身和财产安全，让企业家发挥聪明才智、大胆创新创业。

　　（三）以城乡区域协调发展为方向，全面加快新型城镇化进程。按照“老城提品质、新区出亮点、城镇强特色”的工作思路，分步实施总投资1017亿元的8大类、247项城建工程。加快建设公园城市。完成总投资34.5亿元的省园博园项目，高标准建设全省首个太行文明馆、园林艺术馆等场馆，连通市区东部东关河、南部七里河、北部白马河，形成环城水系，全力办好省第三届园博会和第二届国际城市规划设计大赛，打造山水园博、智慧园博、文化园博的经典传世之作。继续推进太行山前湿地群规划建设，谋划启动鹿城遗址公园等7处郊野公园建设，新建3个城市公园、10个街头游园，厚植“绿满邢襄、水润古城”生态底色。增强城市服务功能和承载能力。市区新改扩建32条道路，启动6条断头路贯通、4条道路改造提升、4座桥梁及3处立体过街通道建设，实施10条小街巷整治，谋划钢铁路与邢衡高速连接线工程，邢台机场年内开工，零换乘客运总站主体完工，构建畅通交通格局。推进邢东新区“五中心一场馆”建设，加快金融中心一期工程，启动会展中心、商务中心、市民中心建设，谋划游客集散中心、新郭守敬纪念馆建设，打造城市发展新高地。围绕城市发展轴线，实施红星美凯龙等一批商业综合体项目，培育壮大总部经济、楼宇经济、会展经济。改造老旧小区203个，保持房地产市场平稳健康发展。常态化开展“美我家乡·文明邢襄”主题创建活动，逐步补齐文明城市创建短板。启动历史文化旅游核心展示区、工业遗址保护等工程，留住牛城记忆。提升城市智能化管理水平。深化与华为公司合作，实施“云上邢台”三年发展计划，加快华为大数据产业园建设，推动各相关市直部门迁移上云，打破部门和行业之间信息孤岛，实现城市智慧式管理和运行。提升市区主要路口交通信号灯智能化水平，有效缓解道路交通拥堵。启动智慧停车平台建设，科学合理设置市区机动车道路停车泊位。严格实行“网格化”管理，共同维护文明有序的市容环境。实施城镇建设攻坚。开展县城建设三年提升行动，组织实施县城建设观摩拉练，研究制定县城高质量发展评价标准。坚持产城教融合发展，统筹建设文教体卫等公共服务设施，提高县城建设品位，加快人口聚集。以省政府确定的12个重点培育特色小城镇为抓手，建设一批独具特色、设施完善的小城镇。

　　（四）以实施乡村振兴战略为总抓手，做好新时代农业农村工作。坚持农业农村优先发展，对标全面建成小康社会必须完成的硬任务，扎实推进乡村振兴。优化农业供给结构。大力发展科技农业、绿色农业、品牌农业、质量农业，加强农田水利基本建设，聚焦农业产业规划、标准质量等关键环节，着力增加农产品有效供给。坚持“藏粮于地、藏粮于技”，确保粮食安全，总产稳定在470万吨左右。加快发展现代都市型农业和特色高效农业，壮大蔬菜、果品、畜牧等优势特色产业，调优蔬菜面积3万亩，发展和改造提升果树面积20万亩。促进农村一二三产业融合，实施农产品加工业提升行动，新增市级重点龙头企业30家以上，以龙头企业为引领，创建一批乡村振兴示范区。支持威县、巨鹿创建国家有机产品认证示范区，鼓励金沙河等争创省政府质量奖。改善农村人居环境。健全多元投入机制，促进城乡基础设施一体规划、互联互通，推动公共服务向农村延伸，社会事业向农村覆盖。深入实施农村人居环境整治三年行动，推进垃圾污水处理、厕所革命、村容村貌提升、四好农村路建设，改造农村无害化卫生厕所20万座以上，新改建农村公路420公里。实施好《邢台市城乡生活垃圾处理一体化管理条例》，山区和平原县（市、区）行政村生活垃圾收集处理率分别达到90%和95%。坚持梯次推进、动态升级，建设一批宜居宜业宜游的美丽乡村。激发农村发展活力。做好农村承包地确权登记颁证后续工作，全面完成集体资产清产核资任务，稳慎推进农村宅基地“三权分置”改革。坚决清理整治“大棚房”，构建常态长效管理机制。推动农业经营体系创新，抓好农民合作社、家庭农场等农村新型经营主体建设。培育新型职业农民，鼓励大学生返乡创业，培养一批农业职业经理人、乡村工匠、文化能人。推进农村移风易俗，培育文明家风、良好乡风、淳朴民风。

　　（五）以改革开放协同创新为突破，增强发展活力动力。坚持改革不停顿、开放不止步，把科技创新摆在更加重要的位置，为高质量发展提供持续动力。大力推进“放管服”改革。着眼于优化营商环境，深入开展“双创双服”，推进“互联网＋政务服务”，巩固提升“两不见面”改革成果，市县90%以上政务服务事项实现“一网通办”，70%以上政务服务事项实现“一窗”分类受理，100个高频事项实现“最多跑一次”。基本完成工程建设项目审批流程再造，大幅度压减审批时限。县级政务服务事项全部进驻综合性政务大厅，市级做到“应进必进”。全面深化重点领域改革。推进国有企业混合所有制和薪酬制度改革，稳妥实施市属经营性国有资产集中统一监管。深化财税金融体制改革，全面实施预算绩效管理，支持县级农信社改制为农村商业银行。深化科技体制改革，创新科研经费使用管理方式，推动产业链、创新链、金融链、政策链深度融合。加快公立医院综合改革，健全疫苗监管制度，推动基本医疗保险支付方式改革，确保医保基金安全。支持威县省级综合改革试点拓展深化，争创更多新经验、新亮点。扩大高水平开放。抢抓京津冀协同发展、雄安新区规划建设、冬奥会筹办等国家战略机遇，聚焦长三角、珠三角、港澳台等资源外溢区域，大力开展总部招商、以商招商、优质股权招商、专业化小团组靶向招商，加快引进一批体量大、质量高、带动强的产业链关联项目。组织开展好百家知名食品企业进邢台、健康食品博览会等活动，叫响“邢字号”招牌。实施“国际市场开拓年”活动，借助“一带一路”，推动钢铁、玻璃、光伏、羊绒等具有比较优势的行业和企业“走出去”。创新开发区体制机制，加快京津产业园、中外产业园发展，支持邢台高新区争创国家级高新区，全面提升开发区高质量发展能级。实施科技创新驱动战略。推进高新技术企业和科技型中小企业双倍增，引导推动优势科技资源和创新服务向企业聚集，力争新增高新技术企业60家、科技型中小企业900家以上。实施创新创业平台扩量提质工程，加强省级以上创新平台建设，新认定省级孵化器1家、省级众创空间2家。深化与北京航空航天大学、武汉理工大学等科研院所合作，加快科技研发成果转化应用。引导企业加大研发投入，进一步提升研发支出占生产总值比重。更加重视人才对高质量发展的智力支撑作用，培育引进一批高端产业创新创业团队以及创新英才，让邢台成为有志之士施展才华、成就梦想的舞台。

　　（六）以解决突出问题为重点，坚决打好三大攻坚战。坚持目标导向、问题导向，聚焦聚力重点战役，全力攻坚，务求实效。

　　打好防范化解重大风险攻坚战。稳妥有序化解金融风险，加强诚信邢台建设，严厉打击非法集资、逃废债等行为，坚决守住不发生系统性区域性金融风险底线。有效防控政府债务风险，落实地方政府限额管理，切实遏制隐性债务增量。深入实施安全生产大排查大整治，全面提升应急管理、应急救援能力和水平，坚决遏制重特大事故发生。持续推进扫黑除恶专项斗争，大力加强公安基层基础建设，扎实开展信访稳定暨“化积案、控风险、筑平安”百日会战，全面做好食品药品安全、动物疫病防疫等各项工作，维护社会大局和谐稳定。

　　打好精准脱贫攻坚战。聚焦“两不愁三保障”，严格落实各项扶贫政策，用好“三包一联”帮扶机制，着力提高扶贫脱贫质量，确保所有贫困县脱贫摘帽，剩余26个贫困村脱贫出列，2.1万贫困人口稳定脱贫。提升产业扶贫实效，统筹推进就业科技生态扶贫，增强贫困地区、贫困群众内生动力和自我发展能力。完成全市易地扶贫搬迁工程。规范扶贫资金管理使用，深化扶贫领域腐败和作风问题专项治理。统筹做好脱贫攻坚“后三年”和“三年后”工作，坚持脱贫不脱政策，对贫困发生率较高的村以及边缘户加强监测预警，防止脱贫人口返贫。

　　打好污染防治攻坚战。加快工业企业清洁生产和超低排放改造，鼓励焦化等企业退城搬迁，“散乱污”企业动态清零；实施清洁取暖改造24.18万户；大幅提高铁路货运比例，提升柴油货车排放达标率，推广新能源车，精细化管控路面污染源，有效应对重污染天气。坚持补水、蓄水、治水并重，发挥好“河长制”作用，“一河一策”加快退污还清，河流考核断面全部达标，地表水劣V类水体比例控制在75%以内。完成大沙河综合治理筑堤固堤工程，适时启动白马河综合治理。编制大运河邢台段专项规划。全面实施“土十条”，加大农用地、建设用地污染风险管控，严格危险废物监管，促进土壤资源永续利用。实施山水林田湖草系统修复工程，以浅山丘陵区为重点，扎实推进国土绿化行动，力争全市完成营造林50万亩以上。始终保持环境执法高压态势，坚决守住生态红线，加快建设天蓝地绿水秀的美丽邢台。

　　（七）以人民对美好生活的向往为目标，着力保障和改善民生。坚持为民办事、为民解忧、为民谋利，继续实施一批民生实事，让人民群众的生活一年更比一年好。

　　实施就业优先战略。突出做好高校毕业生、农民工、退役军人、下岗转岗人员等重点群体就业，促进农村劳动力多渠道转移就业，开展职业技能培训2.5万人次，城镇新增就业6.6万人。加强就业援助，失业人员、困难人员再就业2.5万人，零就业家庭动态清零。严厉打击恶意拖欠农民工工资行为，强化源头治理，完善工程建设领域农民工工资保证金、预储金、政府应急周转金制度，依法保护农民工劳动所得。

　　提高社会保障水平。探索建立城乡居民基本养老保险待遇确定和基础养老金正常调整机制。城镇低保平均标准提高到7920元/年，农村低保标准提高到4400元/年。完成5家特困供养服务机构改造。为符合条件的贫困残疾人提供基本康复服务、辅助工具以及托养服务补贴。棚改开工建设2564套，基本建成818套；完成4类重点对象涉及的农村危房改造1685户，动态新增的全部列入改造计划。

　　推动教育全面振兴。制定全市教育发展规划，加强教师队伍建设，持续发力打好主城区基础教育攻坚战，启动建设市区中小学、幼儿园29所，邢台一中新校区完成部分主体工程，全市新增标准化学校90所，新改扩建乡村学校校舍5万平方米，国家义务教育发展基本均衡县实现全覆盖。推广“国家学生饮用奶计划”，保障质量安全，扩大覆盖范围。推动邢台学院创建综合性应用型大学，启动校园建设三期工程；继续开展职业教育“十优”学校创建活动，支持特教事业发展。

　　做优卫生健康服务。贯彻实施“健康中国人”行动计划，推进分级诊疗工作，加强基层医疗机构标准化、乡村卫生服务一体化建设，全面推开医养结合、长期护理险。加强全科医生培养，支持社会力量举办全科诊所。完成市人民医院新院区建设。加快全市健康信息平台建设，推进群众健康分类管理。加强中医药强市建设，办好第三届全国中医药文化大会。

　　发展文化体育事业。大力实施邢襄文化弘扬工程，推进文化事业、文化产业新发展，市博物馆力争建成开放，加快市群艺馆、市美术馆改造提升，支持沙河、清河创建省级公共文化服务体系示范区。开展全民健身活动，办好市第十五届运动会，启动邢东新区、七里河2处城市运动公园建设。精心办好国际公路自行车赛、上合组织国家国际象棋公开赛、太行山文化带建设交流会，提升影响力和美誉度。

　　加强国防教育和国防后备力量建设，强化退役军人服务保障，让军人成为全社会尊崇的职业。充分发挥工会、共青团、妇联等人民团体桥梁纽带作用，推动民族团结进步创建工作向纵深拓展。高标准做好第四次全国经济普查工作。继续做好民族宗教、新闻广电、外事侨务、人民防空、气象地震、史志档案、邮政通信、公积金、援疆援藏等工作。

　　三、切实加强政府自身建设

　　各位代表！新时代要有新气象、新作为，必须有新视野、新担当。我们将以人民满意为最高追求，旗帜鲜明讲政治，坚定不移抓发展，以更高标准、更严要求、更实作风提升履职能力和工作水平，不断开创各项工作新局面。

　　（一）提高政治站位。自觉以习近平新时代中国特色社会主义思想为指导，树牢“四个意识”，坚定“四个自信”，做到“两个维护”，始终在政治立场、政治方向、政治原则、政治道路上同以习近平同志为核心的党中央保持高度一致。全面落实意识形态工作责任制。深入贯彻新时代党的建设总要求，把坚持和加强党的全面领导贯穿到政府工作全过程、各领域，以实际行动诠释对党的绝对忠诚。

　　（二）推进依法行政。全面加强法治政府建设，严格落实重大行政决策法定程序和行政执法责任制，加强重点领域政府立法。认真执行市人大及其常委会的决议决定，自觉接受人大和政协监督。加大政务信息公开力度，主动接受舆论监督和社会监督。深入学习宣传和贯彻实施宪法，全面加强法治教育，让尊法学法守法用法成为全社会的共同追求和自觉行动。

　　（三）提升施政能力。把深入调查研究作为基本功，养成坚持学习、深入调研的好习惯，一切从邢台实际出发推动工作。弘扬专业精神，切实提高研究政策、运用政策的能力，用专业素养解决发展难题。树立创新思维，积极运用大数据、物联网等新技术推动政府服务创新。改进群众工作方法，努力在基层创新创造中汲取营养，切实增强推动发展和驾驭风险的综合能力。

　　（四）注重落实成效。坚持崇尚实干、力戒空谈，让改革发展稳定的各项决策部署真正落到实处。加快理顺政府管理体制，以机构改革为动力，全力推动政府工作提速、提质、提效。加强诚信政府建设，说了就要算，定下就要办，提高政府公信力。大力倡导知责明责、守责尽责的担当精神，激发你追我赶、比学赶超的干事创业热情，形成主动作为、竞相发展的浓厚氛围。

　　（五）加强廉政建设。认真落实全面从严治党主体责任，深入推进政府系统党风廉政建设和反腐败工作。高标准落实中央八项规定及其实施细则精神，驰而不息纠“四风”、转作风、树新风。持续抓好巡视反馈意见整改落实，加强联市联县监督工作。加大审计监督力度，着力整治群众身边的“微腐败”，努力建设一支忠诚干净担当的高素质公务员队伍。

　　各位代表！功崇惟志，业广惟勤。让我们更加紧密地团结在以习近平同志为核心的党中央周围，在省委、省政府和市委的坚强领导下，践行新理念，抢抓新机遇，展现新作为，以更加优异的成绩庆祝中华人民共和国成立70周年！
